# The Drug Candidate BGP-15 Delays the Onset of Diastolic Dysfunction in the Goto-Kakizaki Rat Model of Diabetic Cardiomyopathy

**DOI:** 10.3390/molecules24030586

**Published:** 2019-02-07

**Authors:** Mariann Bombicz, Daniel Priksz, Rudolf Gesztelyi, Rita Kiss, Nora Hollos, Balazs Varga, Jozsef Nemeth, Attila Toth, Zoltan Papp, Zoltan Szilvassy, Bela Juhasz

**Affiliations:** 1 Department of Pharmacology and Pharmacotherapy, Faculty of Medicine, University of Debrecen, H-4032 Debrecen, Hungary; bombicz.mariann@pharm.unideb.hu (M.B.); priksz.daniel@pharm.unideb.hu (D.P.); gesztelyi.rudolf@pharm.unideb.hu (R.G.); kiss.rita@med.unideb.hu (R.K.); nora@hollos.net (N.H.); varga.balazs@pharm.unideb.hu (B.V.); nemeth.jozsef@med.unideb.hu (J.N.); szilvassy.zoltan@med.unideb.hu (Z.S.); 2 Division of Clinical Physiology, Faculty of Medicine, University of Debrecen, H-4032 Debrecen, Hungary; atitoth@med.unideb.hu (A.T.); pappz@med.unideb.hu (Z.P.)

**Keywords:** diastolic dysfunction, type 2 diabetes, Goto-Kakizaki, BGP-15, metformin, pioglitazone, echocardiography, endothelial dysfunction

## Abstract

*Background and Aims*: Diabetic cardiomyopathy (DCM) is an emerging problem worldwide due to an increase in the incidence of type 2 diabetes. Animal studies have indicated that metformin and pioglitazone can prevent DCM partly by normalizing insulin resistance, and partly by other, pleiotropic mechanisms. One clinical study has evidenced the insulin-senzitizing effect of the drug candidate BGP-15, along with additional animal studies that have confirmed its beneficial effects in models of diabetes, muscular dystrophy and heart failure, with the drug affecting chaperones, contractile proteins and mitochondria. Our aim was to investigate whether the inzulin-senzitizer BGP-15 exert any additive cardiovascular effects compared to metformin or pioglitazone, using Goto-Kakizaki (GotoK) rats. *Methods*: Rats were divided into five groups: (I) healthy control (Wistar), (II) diseased (GotoK), and GotoK rats treated with: (III) BGP-15, (IV) metformin, and (V) pioglitazone, respectively, for 12 weeks. Metabolic parameters and insulin levels were determined at the endpoint. Doppler echocardiography was carried out to estimate diabetes-associated cardiac dysfunction. Thoracotomy was performed after the vascular status of rats was evaluated using an isolated aortic ring method. Furthermore, western blot assays were carried out to determine expression or phosphorylation levels of selected proteins that take part in myocyte relaxation. *Results*: BGP-15 restored diastolic parameters (e′/a′, E/e′, LAP, E and A wave) and improved Tei-index compared to untreated GotoK rats. Vascular status was unaffected by BGP-15. Expression of sarco/endoplasmic reticulum Ca^2+^-ATPase (SERCA2a) and phosphodiesterase 9A (PDE9A) were unchanged by the treatments, but the phosphorylation level of vasodilator-stimulated phosphoprotein (VASP) and phospholamban (PLB) increased in BGP-15-treated rats, in comparison to GotoK. *Conclusions*: Even though the BGP-15-treatment did not interfere significantly with glucose homeostasis and vascular status, it considerably enhanced diastolic function, by affecting the SERCA/phospholamban pathway in GotoK rats. Although it requires further investigation, BGP-15 may offer a new therapeutic approach in DCM.

## 1. Introduction

With the increasing incidence of diabetes mellitus (DM), the cardiovascular (CV) complications of DM are an emerging problem worldwide that needs to be addressed. The development of heart failure (HF) in type 2 diabetes mellitus (T2DM) patients is considered to be independent from other risk factors, such as hypertension, coronary artery disease (CAD) or myocardial infarction [1]. Although proper glucose management reduces CV risk in DM patients, CV event rate is still more frequent than in healthy population, with a 2-5-fold increase in HF risk [2,3]. Diabetic cardiomyopathy (DCM) is diagnosed as characterized by LV diastolic dysfunction and hypertrophy, with or without systolic abnormalities [4]. There are several mechanisms that contribute to the pathophysiology of DCM, including metabolic alterations, inflammation and oxidative stress [1], which will all cause membrane disturbances, calcium-handling abnormalities and sarco/endoplasmic reticulum Ca^2+^-ATPase (SERCA2a) pump dysfunction [1,5]. These processes may lead to relaxation abnormalities, myocyte apoptosis and CV dysfunction [6].

The Goto-Kakizaki (GotoK) strain is one of the most widely used rat model of type 2 diabetes in preclinical research [7]. GotoK rats exhibit mild hyperglycemia, and impaired glucose tolerance, while being normotensive and non-obese at the same time. Thus, they constitute a valuable means for the specific characterization of CV complications caused by hyperglycemia and diabetes only, without the presence of other risk factors [8]. Moreover, GotoK rats display left ventricle (LV) structural remodeling as early as the prediabetes stage, while later on they develop significant cardiomyopathy and diastolic dysfunction [8,9].

Of all the antidiabetic drugs, metformin is currently recommended by the main clinical practice guidelines collectively to use in the vast majority of T2DM patients. Based on a systemic review of observational studies, metformin decreases the mortality in diabetic patients with congestive heart failure [10], and possesses beneficial pleiotropic effects on vascular status [11,12]. Another particularly interesting agent in the field of DCM therapy is pioglitazone, an insulin-sensitizer from the thiazolidinedione (TZD) class of nuclear peroxisome proliferator-activated receptor γ-agonists, which decreases insulin resistance in the liver and peripheral tissues. Moreover, it also exhibits anti-arteriosclerotic and anti-inflammatory effects [13,14]. Even though members of the TZD family initially were seemingly promising antidiabetic drugs with pleiotropic properties, The European Society of Cardiology (ESC) stated in a recent guideline that this group is not recommended for any patient with heart failure, due to its potential of causing fluid retention [10].

In the last decade, the hydroxylamine derivative BGP-15 (*O*-(3-piperidino-2-hydroxy-1-propyl) nicotinic amidoxime) has increasingly raised scientific interest. The rationale for assessing the potential of BGP-15 here, in a model of early-stage diabetic cardiomyopathy, was related to earlier reports of the molecule, as BGP-15 exhibited insulin-sensitizing properties, protective effects against muscular dystrophy, cardiotoxicity, heart failure and atrial fibrillation [15,16,17]. BGP-15 increased tissue insulin-sensitivity in different animal models of metabolic disorders at 10–30 mg/kg doses [18]. BGP-15 entered clinical phase I–II as an insulin-sensitizer, and proved to increase tissue insulin-sensitivity in 200 mg/day and 400 mg/day doses in human patients. It was proven to be particularly safe, causing no clinical side effects or ECG-disturbances [16,17,18]. Parallel animal experiments have shown that BGP-15 increases the expression of heat shock proteins (hsp-s) in certain cells, thus accelerates the regeneration of m. soleus myocytes after cryolysis in 15 mg/kg dose in CB6F1 mice. BGP-15 increased the rate of regeneration, and also improved the contractile function after damage, by accelerating the re-expression of adult myosin heavy chain (MyHC-II and MyHC-I) isoforms and induction of chaperones [17]. Furthermore, 10 days of BGP-15 treatment greatly improved diaphragm muscle fiber function when administered at a dose of 40 mg/kg per day to Sprague-Dawley rats suffering ventilation-induced diaphragm-dysfunction. The treatment provided a protective effect from myosin post-translational modifications, associated with hsp72-induction and polyADP-ribose polymerase 1 (PARP-1) inhibition, resulting in an improvement of mitochondrial function [19]. In another experiment Sapra et al. showed that BGP-15-treatment did not alter the levels of hsp70 or hsp27 in the myocardial tissue, although it increases the phosphorylation of the insulin-like growth factor-1 receptor (IGF1R) in 15 mg/kg dose and also enhanced the expression of SERCA2a gene, moreover inhibited atrial and B-type natriuretic peptide (ANP and BNP) expression. In these experiments, the heart and lungs of the rats were enlarged with collagen deposits, which was counteracted by BGP-15-treatment, as a possible result of a cardioprotective mechanism resulting from IGF1R phosphorylation [16]. It was also reported that BGP-15 (15 mg/kg per day, oral gavage) improved muscle strength and endurance of dystrophic mdx transgenic mice, reduced fibrosis of diaphragm muscle, and increased maximal SERCA activity in diaphragm homogenates [15].

Based on the aforementioned facts, it is likely that BGP-15 has anti-diabetic properties on one hand, and it may enhance muscle function through various mechanisms. In light of the foregoing, this current study was aimed at investigating the effects of BGP-15 in the Goto-Kakizaki non-obese model of T2DM, comparing the drug candidate to the therapeutic golden-standard metformin, and to the insulin-sensitizer pioglitazone with particular interest on cardiac and vascular parameters, and signaling.

## 2. Results

### 2.1. Metabolic Characterisation of Goto-Kakizaki and Wistar Rats

**Figure 1:**
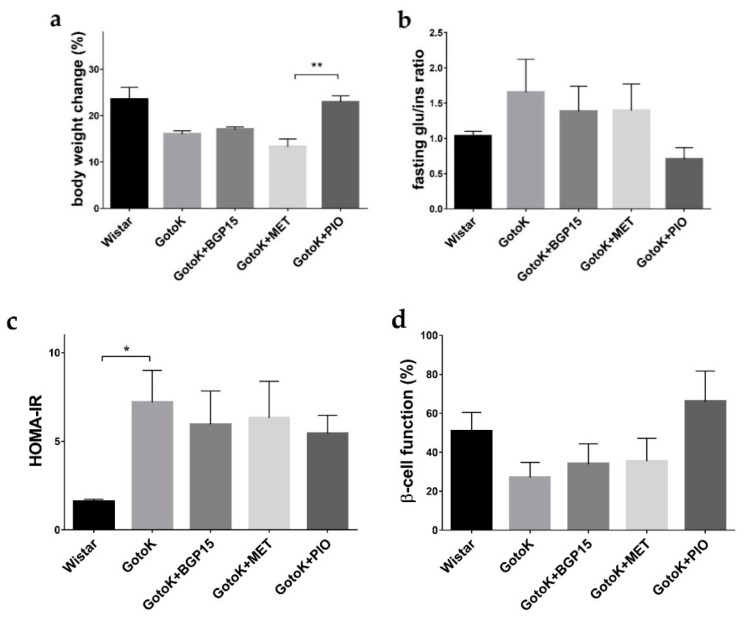
Metabolic parameters in control (Wistar) and diabetic (GotoK) rats treated with vehicle (GotoK), BGP-15 (GotoK + BGP15), metformin (GotoK + MET) and pioglitazone (GotoK + PIO). (**a**) calculated body weight gain in per cent; (**b**) calculated fasting glucose/insulin ratio; (**c**) calculated homeostasis model assessment-estimated insulin resistance (HOMA-IR) index; (**d**) calculated HOMA-B index. Values are presented as mean ±SEM; asterisks (*) denotes significance (Kruskal-Wallis test with Dunn’s post-test, *n* = 6/group, * *p* < 0.05; ** *p* < 0.01).

Table 1 and Figure 1 show serum glucose and insulin levels, as well as the body weight of rat groups (Wistar, GotoK, and GotoK rats treated with BGP-15, metformin and pioglitazone, respectively for 12 weeks). The initial body weights were not significantly different among the four GotoK groups. Despite the random sorting, GotoK and GotoK + MET group showed slightly lower baseline body weights compared to the Wistar group. GotoK groups were roughly resistant to weight gain, with one exception: at the endpoint, Dunn’s post hoc test revealed differences between the MET and PIO group in weight gain. After the 12-week period, fasting glucose level of GotoK rats was significantly higher than those of the Wistar controls, and although all treatments reduced serum glucose, only the pioglitazone treatment was able to exert significant effectiveness (*p* < 0.05). Insulin sensitivity markers were calculated from basal endpoint serum glucose and insulin levels, of which homeostasis model assessment-estimated insulin resistance (HOMA-IR) showed significant differences in GotoK group compared to Wistar (*p* < 0.05).

### 2.2. Endothelium-Dependent Vasorelaxation is Enhanced by Pioglitazone but not BGP-15

In aortic samples of Wistar rats, 10 µmol/L Ach reduced the aortic tension to approximately 50% of the pre-contraction elicited by 10 nmol/L norepinephrine. In the group of GotoK rats without antidiabetic treatment, response to Ach showed a decrease in comparison with that of the Wistar rats (the lack of statistical significance is probably due to the relatively small sample size and the consequent big scatter), thus, the GotoK rats showed impaired endothelial function as compared to the Wistar rats.

**Figure 2:**
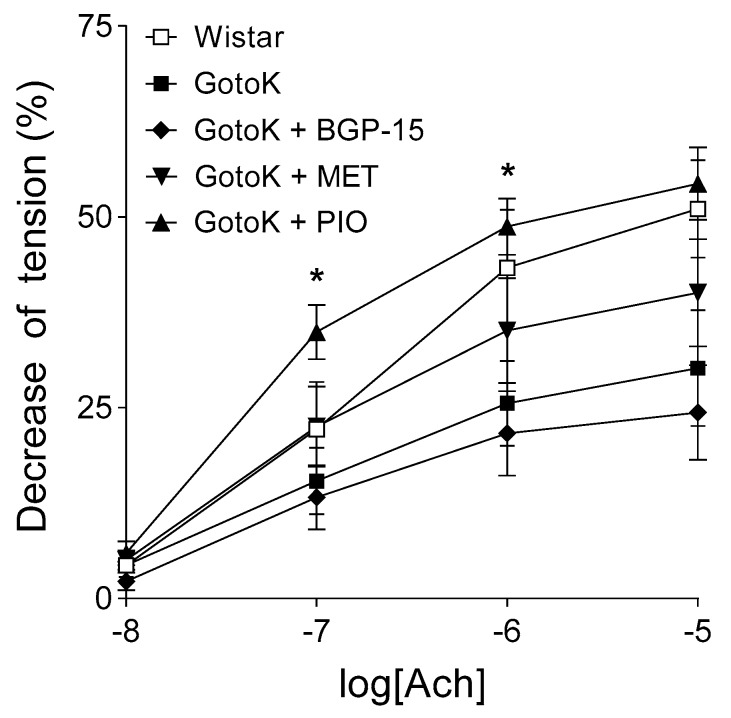
Relaxant effect of acetylcholine (Ach) on the abdominal aorta isolated from Wistar and Goto-Kakizaki rats, and GotoK rats treated orally with BGP-15, metformin or pioglitazone. All aortic rings underwent a pre-contraction elicited by norepinephrine before the administration of Ach. The axis x shows the common logarithm of molar concentration of Ach, while the axis *y* denotes the effect as a percentage decrease of the initial tension of aortic rings. The symbols represent the effect of Ach averaged within the groups (±SEM). Asterisks indicate the significance level of differences between responses to Ach in GotoK and pioglitazone-treated GotoK rats (* *p* < 0.05). *n* = 6/group, one-way ANOVA (with Geisser–Greenhouse correction) followed by Tukey post-testing.

Treatment with BGP-15 and metformin did not significantly improve the deteriorated susceptibility of Goto-Kakizaki rat aorta to Ach, although metformin appeared to enhance the endothelium-dependent arterial relaxation at higher doses. In contrast, pioglitazone considerably increased the response to Ach, that was statistically significant at 100 nmol/L and 1 µmol/L Ach concentrations (when compared to the GotoK group). Moreover, GotoK rats treated with pioglitazone exhibited a greater (*p* = 0.1261 at 0.1 µmol/L Ach) endothelium-dependent arterial relaxation than Wistars (Figure 2).

### 2.3. BGP-15 Enhances Diastolic Function Measured by Echocardiography

**Figure 3:**
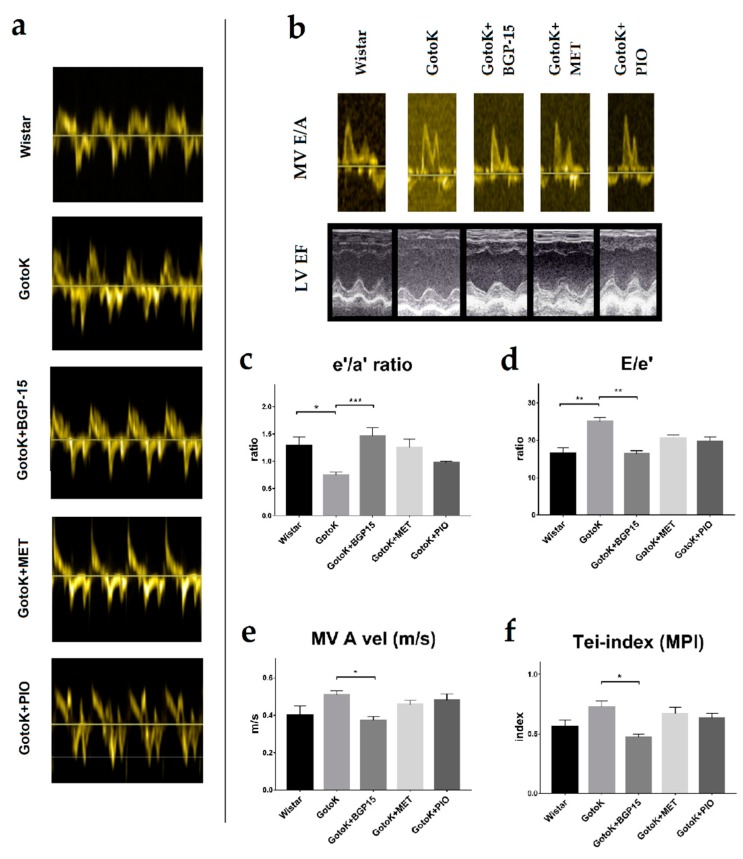
Echocardiographic parameters of rat groups. Data and representative images obtained from healthy control (Wistar) and diabetic GotoK rats treated with vehicle (GotoK), 10 mg/kg BGP-15 (GotoK + BGP15), 100 mg/kg metformin (GotoK + MET) and 10 mg/kg pioglitazone (GotoK + PIO). (**a**) representative images of septal annular tissue velocities (s′, e′ and a′ waves) of rats, recorded by TDI echocardiography; (**b**) representative images of mitral inflow velocities (E and A waves), obtained by Doppler imaging, and representative parasternal long axis views of the left ventricle, obtained by M-mode (EF: ejection fraction); (**c**) graph of septal e′/a′ ratio of treatment groups; (**d**) calculated E/e′ ratios of rats; (**e**) graph of mitral valve (MV) atrial (A)-wave velocities; (**f**) myocardial performance, shown as Tei-index of treatment groups. All data is presented as mean SEM, *n* = 6/group, Kruskal-Wallis test with Dunn’s post-test. Asterisks denote the level of significance (* *p* < 0.05; ** *p* < 0.01).

Diastolic function assessed by echocardiography worsened in GotoK rats compared to Wistars, but was restored in the GotoK + BGP15 group (Table 2 and Figure 3a–f). The ratio of early and late diastolic mitral annular velocities (e′/a′) was reversed in the GotoK group (0.744 ± 0.056, *p* = 0.0386 vs. Wistar), but was elevated significantly in BGP-15-treated rats (1.458 ± 0.155, *p* = 0.0023 vs. GotoK). E/e′ ratio (indicative for LV filling pressure) increased in GotoK rats (*p* = 0.0045 vs. Wistar), but was normalized in GotoK + BGP15 group (*p* = 0.0019 vs. GotoK). Both early (E) and atrial (A) peak mitral filling velocities increased in GotoK (*p* = 0.002, and *p* = 0.1806 vs. Wistar), but decreased in BGP-15-treated group (*p* = 0.0093, and *p* = 0.0499 vs. GotoK). E/A ratios and DecT were not significantly different in GotoK and Wistar groups.

Systolic function was slightly worsened in GotoK group, as left ventricle ejection time (LV ET) was shortened (*p* = 0.05 vs. Wistar), but increased in the GotoK+BGP15 groups (*p* = 0.0001 vs. GotoK), and fractional shortening (FS) showed non-significant decrease (*p* = 0.087 vs. Wistar). Global myocardial performance assessed as Tei-index slightly worsened in the GotoK group (*p* = 0.388 vs. Wistar) and increased significantly in the BGP-15-treated animals (*p* = 0.0147 vs. GotoK).

GotoK rats treated with metformin had preserved annular e′ to a′ ratios, systolic function (fractional shortening, ejection fraction), however, parameters did not reach the level of significance in comparison to GotoK group. Echocardiographic parameters of pioglitazone-treated did not differ significantly from untreated GotoK rats.

### 2.4. BGP-15 Elevates the Phosphorylation Level of Phospholamban and VASP Proteins

**Figure 4:**
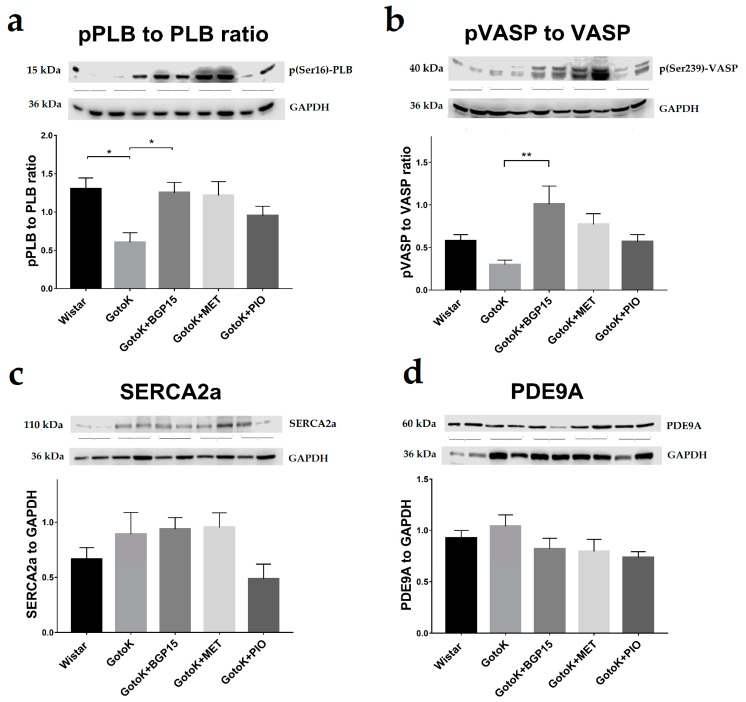
Expression SERCA2a and PDE9A proteins, with pPLB/PLB and pVASP/VASP ratios, obtained by Western blot. (**a**) phospho(Ser16)-phospholamban (pPLB) to phospholamban (PLB) ratios in groups of rats; (**b**) phopsho(Ser239)-vasodilator-stimulated phosphoprotein (pVASP) to vasodilator-stimulated phosphoprotein (VASP) ratios; (**c**) sarcoplasmic/endoplasmic reticulum calcium ATPase 2a (SERCA2a) levels, normalized to GAPDH as a housekeeping protein; (**d**) expression level of phosphodiesterase 9A (PDE9A) levels, normalized to GAPDH. All data is presented as mean ±SEM, data was averaged from 3 independent experiment (*n* = 6/group), Kruskal-Wallis test with Dunn’s post-test. Asterisks denote the level of significance (* *p* < 0.05; ** *p* < 0.01).

Western blot analyses carried out by phospho-specific antibodies (for the serine-16 (Ser16) residue of phospholamban (PLB), and serine-239 (Ser239) residue of vasodilator-stimulated phosphoprotein (VASP)), revealed significant differences in phosphorylation levels of PLB and VASP in myocardial tissues of rat groups (Figure 4a–b). 

The ratio of phosphor(Ser16)-PLB to unphosphorylated PLB (pPLB to PLB ratio) was significantly decreased in the GotoK group in comparison to Wistar (*p* = 0.0171), but increased in the GotoK + BGP15 groups compared to untreated GotoK (*p* = 0.0348). Metformin treatment showed similar trend (*p* = 0.1432, vs. GotoK).

As the serine-239 residue of PLB is a phosphorylation target of Protein Kinase G (PKG), expression of phospho(Ser239)-VASP (indicative for PKG activity) was also measured, and phospho(Ser239)-VASP to unphosphorylated VASP ratios (pVASP to VASP) in each subjects were calculated (Figure 4b). Ratio of pVASP to VASP showed similar trend as those of pPLB to PLB, as the pVASP to VASP ratio significantly increased in the GotoK + BGP15 group in comparison to untreated GotoK (*p* = 0.0094), and also showed non-significant increase in metformin-treated rats (*p* = 0.0877 vs. GotoK).

Expression of SERCA2a was found to be unchanged among treatment groups (Figure 4c). The expression levels of phosphodiesterase 9A (PDE9A; Figure 4d), responsible for the degradation of cyclic-guanosine monophosphate (cGMP) did not differ significantly among groups.

## 3. Discussion

Diabetes incidence is increasing worldwide, and the progression of the disease leads to several life-threatening complications, as diabetes affects most tissues of the human body, causing multi-organ damage. The cardiovascular system is of particular importance, proven by the fact that two-third of diabetes-related mortality is caused by CV events [20]. Diabetes-associated cardiac disease may appear in a form of (1) coronary artery disease (CAD), (2) autonomic neuropathy (CAN), or (3) diabetic cardiomyopathy (DCM), which is thought to be underdiagnosed in several cases, despite its unquestionable importance, in particular regarding the fact that diabetes carries a 2-5-fold increase in heart failure risk, and 30–60% of the T2DM patients have diastolic dysfunction [20,21].

Several mechanisms have been proposed to contribute to the pathological changes in diabetic cardiomyopathy, including: metabolic abnormalities, autonomic dysfunction, inflammation, oxidative stress and subcellular signaling disturbances, demonstrating the complexity of the condition [1]. Among molecular mechanisms, three processes: mitochondrial dysfunction, endoplasmic reticulum (ER) stress and impaired calcium (Ca) handling are particularly important. These abnormalities may be the consequence of oxidative stress caused by increased reactive oxygen species (ROS) production and by the formation of advanced glycation end products (AGEs): damaged and reactive proteins, that are capable of forming cross-links between structural components of the cell, or disturb the function of other proteins and enzymes. AGEs exert detrimental effects once by their biochemical properties, and also by acting via specific receptors (RAGEs), through which transcription factors of proinflammatory genes are activated [1,5]. Increased production of ROS and AGEs cause stress and damage in the endoplasmic reticulum by disturbing protein folding, post-translational modifications and Ca-handling. ER stress causes membrane instability, Ca-handling abnormalities and diminishes the activity of sarcoplasmic/endoplasmic reticulum calcium ATPase (SERCA2a) pump, resulting in lengthened relaxation, impaired diastolic function, myocyte apoptosis and cell death [6]. The deteriorated function of SERCA2a pump was also confirmed in myocardial samples of type 1 diabetic (T1DM), streptozotocin-induced diabetic rats, as a result of hyperglycemia [6]. Furthermore, it was previously shown that nitro-oxidative stress, and apoptosis accompanied by systolic dysfunction are more prominent in T1DM models, while in T2DM, cardiomyocyte hypertrophy, stiffness and diastolic dysfunction are more characteristic [22].

In this study, we attempted to compare the effect of two standard antidiabetic drug (metformin, pioglitazone) with a promising drug candidate, hydroxamic-acid derivative BGP-15. For the sake of completeness, we examined their impact on metabolic parameters as well, even though this current study was aimed to assess the cardiovascular profile of the agents. Here, we confirmed that the GotoK rats had T2DM characterized by mild hyperglycemia and modest hyperinsulinemia without weight gain [9]. Which concerns of glucose homeostasis, GotoK rats showed the signs of insulin resistance to the end of 12-week-long treatment. Fasting glucose level was significantly elevated, while fasting insulin was slightly increased compared to the values of the control group. To further confirm the insulin resistance in GotoK model, HOMA-IR were calculated from basal glucose and insulin levels, as a widely used insulin sensitivity marker [23]. Basal glucose and HOMA-IR were significantly elevated in GotoK rats, but decreased after pioglitazone-treatment, moreover, improved NO-dependent endothelial function was measured in the GotoK+PIO group. In accordance with our results, several recent studies indicate that thiazolidinediones are able to improve endothelial function, yet the exact mechanisms are yet to be determined [24,25]. In fact, the exact pathophysiological processes by which insulin resistance contributes to endothelial dysfunction are mysterious as well, despite the fact that the last two decades of research have established the crucial role of endothelial cells in defense against metabolic syndrome [26]. On the other hand, in our setting, pioglitazone-treated animals exhibited marked weight gain, especially in comparison with the metformin-treated group. Besides this one exception, there were no significant differences in body weight change among treatment groups. As long as GotoK is a non-obese spontaneous T2DM model, weight gain can be regarded as an adverse event [27]. It is believed by that the weight gain is the result of water retention, yet Hallakou et al. described that pioglitazone stimulates adipocyte differentiation in vivo and induces an increase in glucose utilization in the adipose tissue by increasing the expression of insulin-responsive GLUT, fatty acid synthase, and phosphoenolpyruvate carboxykinase genes [28,29]. Additionally, body-weight-increasing action of pioglitazone is a disadvantage in diabetic patients, since obesity aggravates diabetes and promotes cardiovascular diseases and atherosclerosis [30]. According to our knowledge, weight gain is the major problem that may limit the therapeutic value of pioglitazone [31]. Moreover, a recent meta-analysis showed that although pioglitazone-treatment is associated with reduced risk of non-fatal CVD events and insulin-resistance, it may increase the risk of weight gain, edema and heart failure [32].

Echocardiographic parameters presented here show marked differences between treatment groups. Untreated GotoK rats exhibited mild diastolic dysfunction demonstrated by reversed tissue e′/a′ ratios, elevated E/e′ ratios (indicative for LV filling pressure) and mitral A wave, compared to age-matched Wistar animals. With E/A ratios higher than 1, increased A wave, reversed e′/a′ and highly elevated E/e′ ratios, untreated GotoK rats showed similar pattern of diastolic dysfunction to the nominal “pseudonormal” or Grade 2 diastolic dysfunction described in guidelines for human patients [33]. Metformin treatment did not delay the onset of diastolic dysfunction, although some parameters (e′/a′, FS) showed trend towards increase, and endothelium-dependent vasorelaxation was slightly improved, in accordance with findings of other investigators, who previously showed that metformin reduces oxidative stress, thus improves endothelial function in diabetic rats [11]. Serum glucose levels of metformin-treated animals only showed a non-significant decrease in comparison to GotoK rats. The effectiveness of metformin in reducing blood glucose of GotoK rats is controversial in literature. Sena et al. showed that MET decreases blood glucose even at 60 mg/kg dose in GotoK rats [11], while Tae et al. showed that metformin failed to reduce baseline serum glucose levels of GotoK rats, and only improved glucose tolerance in OGT tests, even in much higher (300 mg/kg) doses [34]. As GotoK rats are insulin-resistant to some extent and have reduced beta cell mass [35], additionally, metformin primarily decreases plasma glucose by activating its utilization in the GotoK rat [36], we assume that in our setting, the relative ineffectiveness of metformin may be the result of the smaller dose (100 mg/kg) or the relatively small (*n* = 6) number of tested animals. Pioglitazone, although reduced serum glucose and restored endothelium-dependent vasorelaxation, failed to significantly improve echocardiographic parameters in this experiment, which may partly be associated with weight gain.

Notwithstanding, echocardiographic examination BGP-15-treated rats revealed that the treatment restored diastolic parameters, and also improved myocardial performance in GotoK animals, despite the fact that it failed to improve endothelial function, or significantly reduce blood glucose levels in our experimental setting. Transmitral Doppler revealed normal E/A ratios, and significantly improved E and A velocities compared to GotoK rats. Transmitral Doppler alone however is not enough to properly assess and classify the grade of diastolic dysfunction, and needs to be complemented with other decisive approaches, such as Tissue Doppler Imaging (TDI), a more sensitive method in evaluating chamber relaxation and estimating pressure relationships [37]. Here, we show that tissue e’/a’ ratio was significantly increased in BGP-15-treated rats, and E/e’ ratios were significantly lower in comparison to diseased GotoK. Ratio of E/e’ is a reliable indicator of LV filling pressure, additionally, echocardiographic estimation of filling pressure may provide similar information to invasive cardiac catheterization, making it an attractive way to diagnose relaxation abnormalities [38]. Accordingly, determination of E/e’ ratios allows us to estimate the left atrial pressure (LAP). LAP values of BGP-15 treated rats showed similar pattern to E/e′ ratios, and decreased significantly (to the control level) in comparison to GotoK rats. Besides diastolic parameters, BGP-15 treatment also improved other variables, as LV ejection time (LV ET) significantly increased in GotoK+BGP15 group, showing more proper ejection in systole, and Tei-index—indicative of global myocardial performance—was also significantly improved in BGP-15-treated animals compared to untreated GotoK.

One possible explanation for echocardiographic findings is the hypothesis that BGP-15 treatment may enhance relaxation properties of myocytes via affecting the activity (phosphorylation) of small regulatory proteins, that take part in controlling sarcoplasmic/endoplasmic reticulum calcium ATPase (SERCA2a) pump function. SERCA2a pump, a significant determinant of diastolic function, is regulated by phosphorylation of phospholamban (PLB) protein, which increases SERCA2a activity in its phosphorylated state (pPLB) on its Ser16 or Thr17 residues [39]. Phospholamban may be phosphorylated on its Ser16 by the cGMP-dependent Protein Kinase G (PKG, also known as cGKI), resulting in enhanced relaxation and increased lusitropy of the myocardium [39,40]. Here, phospho-specific antibodies were used to determine the phosphorylation state of PLB, and analyzes showed marked differences among treatment groups, as pPLB to PLB ratios were diminished in the diseased GotoK animals compared to healthy controls. On the contrary, increased pPLB to PLB ratios were found in the subjects of BGP-15-treated group. This finding suggests that the activity of the SERCA pump increased in the GotoK+BGP-15 group, which may contribute to enhanced relaxation of the myocytes, thus restoring diastolic parameters of the heart, confirmed by Doppler echocardiography. A limitation of this current report is that myocyte force/contraction measurements or calcium-sensitivity assays, which would further strengthen our findings, were not carried out.

Recent evidence suggests the crucial role of PKG in myocyte calcium homeostasis through SERCA pump regulation. In an elegant experiment Frantz et al. showed that mice with cardiomyocyte-restricted PKG deletion exhibited reduced phosphorylation of PLB on Ser16 and diminished SERCA activity, resulting in deteriorated cardiac function, additionally, it was also shown that PLB Ser16 is the specific target for PKG [41,42]. Based on these data, we attempted to detect PKG activity in our experimental setting, by examining phosphorylation of VASP protein, using the phospho(Ser239)-specific 16C2 antibody. Ser239 residue of VASP is a unique phosphorylation target of PKG, thus measuring VASP phosphorylation a widely accepted method to indirectly assess the activity of cGMP-dependent Protein Kinase G [43]. Here, we showed that along with increased phosphorylation of phospholamban in the BGP-15-treated group, the pVASP to VASP ratios were also elevated, suggesting increased activity of the PKG enzyme, which may explain the phosphorylation of both downstream targets (PLB and VASP), manifested in improved diastolic parameters of the heart. What concerns myocardial cGMP, is a limitation of this report that we did not directly measure cGMP levels, instead, we attempted to examine the expression of the cGMP-degrading enzyme PDE9A. Recent evidence suggests the role of PDE9A in regulation of natriuretic-peptide-derived cGMP levels and thus activity of PKG, further, upregulation of PDE9A was previously shown in cardiac dysfunction (traverse aortic constriction and atherosclerotic disease) models by others and our research team as well [44,45]. Based on the aforementioned data, we determined PDE9A levels in myocardial samples of animals, however, we failed to show significant differences between the treatment groups in this experimental setting. As crosstalk between PDEs, and enhancement of the cGMP-system in cardiovascular disorders are of intense interest, several questions raised here will presumably be addressed in further studies.

Regarding study limitations and potential bias, it needs to be addressed, that we were not able to undoubtedly confirm the insulin-sensitizing effect of BGP-15. Although blood glucose levels and HOMA-IR of BGP-15-treated rats were lower in comparison to GotoK, the difference was not significant, possibly due to the relatively small sample size, or the strength of the statistical probe. From our presented results, it may be assumed, that BGP-15 enhances PLB/SERCA pathway through the Protein Kinase G system. To reveal the mechanism of action of BGP-15, these experiments needed to be completed using other techniques. In addition, investigating the effects of BGP-15 on healthy animals would further strengthen our results and support the precise characterization of the drug candidate.

In conclusion, in this report our aim was to examine the cardiovascular complications solely caused by T2DM and to evaluate the ability of the drug candidate BGP-15 in restoring diastolic heart functions, by comparing the agent with classical antidiabetics with proven pleiotropic effects. BGP-15 restored the diastolic dysfunction observed in untreated animals, elevated the phosphorylation level of VASP and phospholamban proteins, without a significant effect on vascular function or serum glucose in this experimental setting. Since two-third of diabetes-related mortality is caused by cardiovascular events, and BGP-15 is protective in DCM, therefore, we assume that it would be promising to examine the combined effects of BGP-15 and classical antidiabetics in reducing disease complications. Nevertheless, further investigations are needed to identify cellular targets and the clinical value of the drug candidate in diabetic cardiomyopathy.

## 4. Materials and Methods

### 4.1. Animal Model and Study Design

The present study was approved by the local Ethics Committee of University of Debrecen and the animals received humane care in accordance with the “Principles of Laboratory Animal Care” by EU Directive 2010/63/EU (permission: 25/2013DEMÁB; 29 January 2014). 12 week-old Goto-Kakizaki (GotoK) and Wistar (wild-type) rats were used in this present study, and rats were housed in a controlled temperature room with a 12:12 dark:light cycle. 

**Figure 5:**
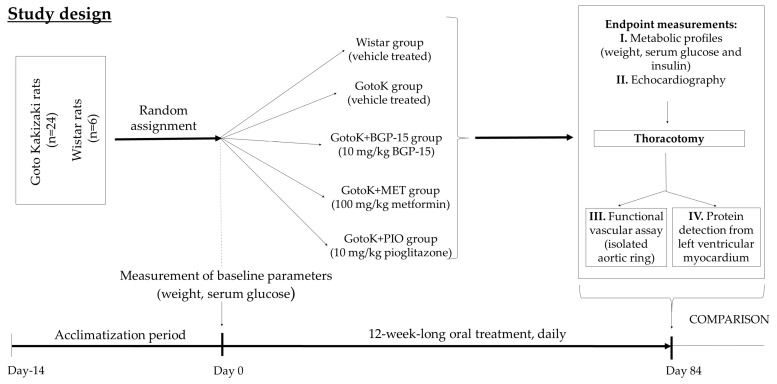
Flow-chart of study design. After acclimatization, healthy control (Wistar) and diabetic GotoK rats were randomly divided into subgroups, as follows: (I) Wistar control, treated with vehicle; (II) diseased GotoK treated with vehicle (GotoK), (III) GotoK treated with 10 mg/kg BGP-15 (GotoK + BGP15), (IV) GotoK trated with 100 mg/kg metformin (GotoK + MET), and (V) GotoK treated with 10 mg/kg pioglitazone (GotoK + PIO), for 12 weeks. At the endpoint, echocardiography was carried out, serum parameters were measured, and after thoracotomy, aortic rings were cut off for ex vivo experiments, and left ventricle myocardium was frozen for further analyses (Western blot).

Rats were randomly divided into five subgroups (*n* = 6/group): Wistar group (served as control group, vehicle-treated); diseased GotoK group (GotoK rats, vehicle-treated); GotoK + BGP15 group (GotoK rats treated daily with 10 mg/kg BGP-15, per os); GotoK+MET group (GotoK rats receiving 100 mg/kg metformin daily, per os) and GotoK + PIO group (GotoK rats treated daily with 10 mg/kg pioglitazone, per os), for 12 weeks (Figure 5). All animals were executed under deep thiopental anesthesia (50 mg/kg, i.p.), then hearts were excised rapidly, and frozen in liquid N_2_, while aortic samples were subjected to ex vivo functional experiments.

### 4.2. Chemicals

**Figure 6:**
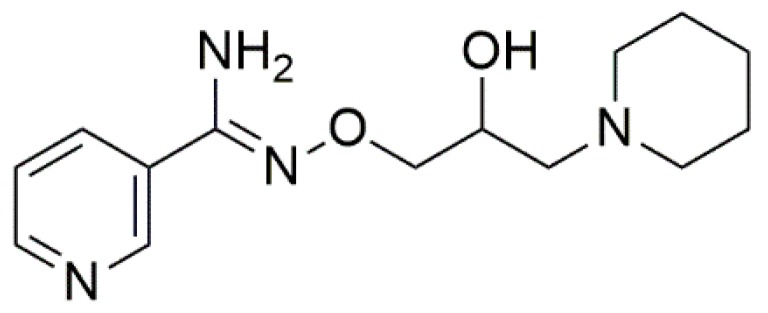
Chemical structure of *O*-(3-piperidino-2-hydroxy-1-propyl)-nicotinic amidoxime, known as BGP-15, a hydroxamic-acid derivative small molecule (the figure was made using ChemDraw Ultra software ver.12., CambridgeSoft, Perkin Elmer, Waltham, MA, USA).

Chemicals, buffers, primary and secondary antibodies, and ingredients of Krebs solution were obtained either from Sigma-Aldrich-Merck KGaA (Darmstadt, Germany), and Abcam Plc. (Cambridge, UK). Norepinephrine hydrochloride (NE; as Arterenol^®^), acetylcholine chloride (Ach), adenosine 5′-triphosphate disodium salt hydrate (ATP), metformin hydrochloride and pioglitazone hydrochloride were purchased from Sigma-Aldrich-Merck KGaA. BGP-15 (Figure 6) was obtained from N-Gene Research Laboratories (Budapest, Hungary).

### 4.3. Metabolic Profiles

Body weight of animals were measured weekly, fasting blood glucose and plasma insulin levels were determined at the endpoint of the experiment. Blood was collected from tail vein, and glucose levels were determined using an Accu-Chek Active blood glucose meter (Roche Diagnostics, Mannheim, Germany). After, blood samples were centrifuged (Centrifuge 5415R; Eppendorf AG, Hamburg, Germany) for 2 min at 4 °C and 10.000 rpm; then plasma was aliquoted, frozen, and stored at −80 °C for subsequent determinations of basal plasma insulin level. Plasma insulin levels were determined using a commercially available radioimmunoassay kit (RK-400CT, Institute of Isotopes of the Hungarian Academy of Sciences, Budapest, Hungary) [46]. The homeostasis model assessment of insulin resistance (HOMA-IR) was calculated as follows: Fasting insulin (μIU/ml) × fasting glucose (mmol/mL)/22.5; and β-cell function (HOMA-B) was calculated using the following formula: 20 × fasting insulin (μIU/mL)/fasting glucose (mmol/mL) − 3.5 [47].

### 4.4. Echocardiography

**Figure 7:**
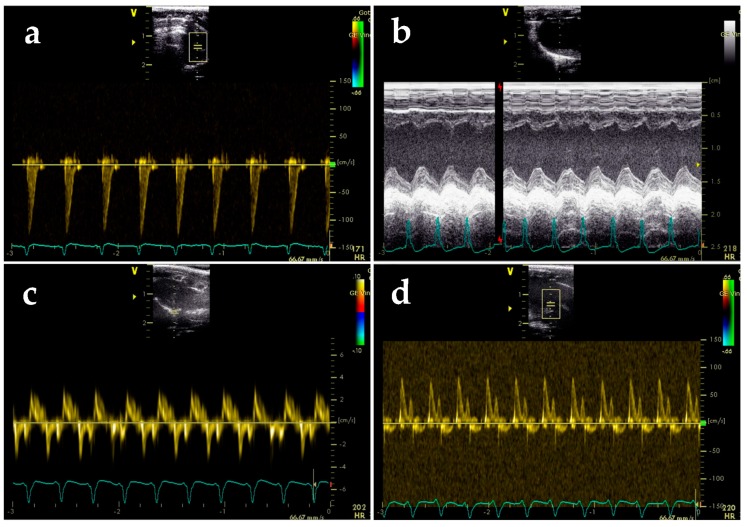
Representative images of echocardiographic experiments. (**a**) Left-ventricle outflow tract (LVOT), visualized by Doppler echocardiography; (**b**) parasternal long axis, M-mode image of left ventricle; (**c**) 3-chamber view, mitral annular velocities, tissue Doppler mode; (**d**) mitral inflow velocities, visualized by Doppler echocardiography.

Echocardiography was carried out by a Vivid E9 sonograph with an i13L linear-array probe (GE Healthcare, New York, NY, USA). Rats were anaesthetized with ketamine/xylazine combination (75/5 mg/kg, respectively), chest hair was shaved and animals were positioned in a dorsal position. Data acquisition was performed in 2D-, M-, and Doppler modes, from parasternal long- and short axis, as well as apical 4 chamber views (Figure 7). Cardiac function was assessed in accordance with the guideline of American Society of Echocardiography. Wall thicknesses, and chamber diameters were measured in M-mode loops, at mid-level of the papillary muscles, from both parasternal long axis and short axis views. Left ventricle internal diameter in diastole (LVIDd) and in systole (LVIDs), as well as wall thicknesses were measured by standard methods. Ejection fraction was calculated as EDV-ESV/ESV end-diastolic volume (EDV, Teiholz-formula). Diastolic function was assessed by Doppler and Tissue Doppler imaging from apical 3- and 4 chamber views. Mitral valve closure to opening time (MCOT), isovolumic contraction time (IVCT), ejection time (ET) and isovolumic relaxation time (IVRT) were determined from tissue Doppler (TDI) frames. Myocardial performance index was calculated as Tei-index (IVRT+IVCT/LVET). Data analysis was carried out using EchoPAC PC software ver. 112, (GE Healthcare, New York, NY, USA), by a blinded reader. Three cardiac cycles were averaged for each parameter, and data is presented as mean ±SEM.

### 4.5. Functional Vascular Assay

After sacrificing the rats, the proximal part of the abdominal aorta was isolated, and 2 mm wide rings were cut off (two rings from each animal). The rings were mounted horizontally at 10 mN resting tension, using wire instruments, in 10-mL vertical organ chambers (Experimetria TSZ-04, Experimetria Ltd, Budapest, Hungary). The chambers contained Krebs solution oxygenated with 95% O_2_ and 5% CO_2_ (36 °C; pH = 7.4). The isometric contractile force of the circulatory muscle layer of aortic rings was measured by a transducer (Experimetria SD-01,Experimetria Ltd, Budapest Hungary) and strain gauge (Experimetria SG-01D), and recorded by a polygraph (Medicor R-61 6CH Recorder, Medicor, Hungary).

After a 60-min incubation period, 10 nmol/L norepinephrine was administered to the rings (a norepinephrine concentration near to the half maximal effective concentration (EC_50_) for Wistar rat aorta; [48]). After the stabilization of the contractile force (pre-contraction), a cumulative Ach E/c curve was constructed (from 10 nmol/L to 10 µmol/L).

Responses of aortic rings obtained from the same rat were averaged. The effect of Ach was defined as a percentage decrease in the pre-contraction produced by norepinephrine (in addition to the resting tension).

Normality of data sets was verified with Shapiro-Wilk test. Data sets (more than two) were compared using one-way ANOVA (with Geisser–Greenhouse correction) followed by Tukey post-testing. Difference of means was considered significant at *p* < 0.05. Statistical analysis was carried out with GraphPad Prism 7.04 (GraphPad Software Inc., La Jolla, CA, USA).

### 4.6. Western blot

Proteins from left ventricular (LV) myocardial tissues were identified by western blot analysis. For protein extraction, 300 mg from the deep-frozen samples (at −80 °C) were placed in liquid nitrogen and grounded gently into a fine powder. The powdered tissues were homogenized in 800 µL Buffer (25 mM Tris-HCl, pH = 8, 25 mM NaCl, 4 mM Na-orthovanadate, 10 mM NaF, 10 mM Na-pyrophosphate, 10 nM okadaic acid, 0.5 mM EDTA, 1 mM PMSF and protease inhibitor cocktail (Sigma-Aldrich, St. Louis, MO, USA)) by a Polytron-homogenizer (IKA-WERKE, Staufen, Germany), and samples were then centrifuged at 10.000 g for 20 min. Total Protein concentration of the supernatant was determined using a Bicinchoninic Acid Protein Assay Kit (QuantiPro™ BCA Assay Kit, Sigma-Aldrich-Merck KGaA, Darmstadt, Germany). Samples containing 20 µg total protein were separated using 10, 12 or 18% SDS-PAGE, according to the molecular size of the examined protein. After gel electrophoresis at 40 mA for 100–120 min, proteins were transferred onto a nitrocellulose membrane (GE Heathcare, New York, NY, USA) via electro-blotting at 25 V for 90 min. The membranes were incubated in TBS-T containing 3% BSA for 1 hour at room temperature, then were probed overnight at 4 °C with the following primary antibodies: anti-phospholamban (PLB), anti-phospho(Ser16)-phospholamban (pPLB), anti-sarcoplasmic/endoplasmic reticulum calcium ATPase 2a (SERCA2a), anti-vasodilator-stimulated phosphoprotein (VASP), anti-phopsho(Ser239)-vasodilator-stimulated phosphoprotein clone 16C2 (pVASP), anti-phosphodiesterase 9A (PDE9A) and anti-glyceraldehyde-3-phosphate-dehydrogenase (GAPDH), obtained from Sigma-Aldrich (Sigma-Aldrich-Merck KGaA and Abcam (Abcam Plc., Cambridge, UK). All primary antibodies were used as recommended by the manufacturer. After, membranes were incubated with secondary antibodies labeled with horseradish peroxidase. Proteins were developed with enhanced chemiluminescense reagent (WesternBright™, ECL, Advansta Inc., Menlo Park, CA, USA) and the detection was accomplished by a C-Digit^®^ blot scanner with Image Studio Digits ver. 5.2. software (LI-COR Inc., Lincoln, NE, USA) [45]. Data was averaged from 3 independent experiment (*n* = 6/group), and statistical analysis was carried out using Kruskal-Wallis test with Dunn’s post-test.

### 4.7. Statistical Procedures

Statistical analyses were performed by GraphPad Prism software for Windows, version 7.01 (La Jolla, CA, USA). All data are presented as the average outcome in a group (mean) ± standard error of the mean (SEM). The D’Agostino-Pearson normality test was used to estimate Gaussian distribution. Kruskal-Wallis test with Dunn’s post-test was used to estimate differences between groups with non-Gaussian distribution. Probability values (*p*) less than 0.05 were considered significantly different.

## 5. Conclusions

Diabetic cardiomyopathy is an emerging problem worldwide as the incidence of type 2 diabetes is increasing. Here, we evaluated the effectiveness of BGP-15 in alleviating cardiac dysfunction in the Goto-Kakizaki rat model of diabetic cardiomyopathy, and compared the agent with classical antidiabetics with proven pleiotropic effects. BGP-15-treatment restored the diastolic dysfunction observed in untreated animals, increased the phosphorylation level of the SERCA-regulator phospholamban and the Protein Kinase G target VASP protein, without significant effects on vascular function or glucose homeostasis. We assume that BGP-15 improves diastolic function partly by enhancing the activity of the phospholamban/SERCA pathway.

## Figures and Tables

**Table 1:** Fasting serum glucose, fasting plasma insulin levels, and body weights before (baseline) and after (endpoint) the treatment.

Parameter	Fasting Glucose (mmol/L)	Fasting Insulin (μIU/mL)	Baseline BODYWEIGHT (g)	Endpoint Bodyweight (g)
Wistar	6.280 ± 0.428	5.850 ± 0.132	407.8 ± 3.798	539.8 ± 22.45
GotoK	13.62 ± 0.626 *	12.53 ± 3.292	313.8 ± 4.070 *	374.0 ± 6.266 *
GotoK+BGP15	10.92 ± 0.561	12.30 ± 3.793	318.3 ± 5.566	383.8 ± 9.024
GotoKMET	11.20 ± 1.002	12.82 ± 4.104	312.7 ± 5.655 *	361.7 ± 12.27 *
GotoK+PIO	7.880 ± 0.312 ^#^	14.83 ± 2.768	316.3 ± 3.658	411.3 ± 10.40

Metabolic parameters were determined in control (Wistar) and diabetic (GotoK) rats treated with placebo (GotoK), BGP-15 (GotoK + BGP15), metformin (GotoK + MET) and pioglitazon (GotoK + PIO). Values are presented as mean ±SEM, *n* = 6/group, Kruskal-Wallis test with Dunn’s post-test. * vs. Wistar; ^#^ vs. GotoK, (*p* < 0.05).

**Table 2:** Echocardiographic parameters of rats, obtained by 2D, M-mode, Doppler (PW) and tissue Doppler (TDI) imaging at the endpoint of the study.

Parameter	Wistar	GotoK	GotoK + BGP-15	GotoK + MET	GotoK + PIO
LVIDd (mm)	7.267 ± 0.278	6.918 ± 0.167	7.243 ± 0.183	6.483 ± 0.425	7.323 ± 0.400
LVIDs (mm)	4.365 ± 0.201	4.515 ± 0.095	4.69 ± 0.193	3.940 ± 0.200	4.478 ± 0.345
FS (%)	39.94 ± 1.474	33.27 ± 1.667	37.62 ± 0.727	38.85 ± 1.715	39.21 ± 1.608
EF (%)	68.92 ± 1.813	60.36 ± 2.343	66.13 ± 0.891	67.94 ± 2.068	67.98 ± 2.110
MV E vel. (m/s)	0.612 ± 0.047	0.883 ± 0.024 **	0.648 ± 0.021 ^##^	0.763 ± 0.025	0.810 ± 0.033
MV A vel. (m/s)	0.402 ± 0.048	0.507 ± 0.023	0.372 ± 0.022 ^#^	0.457 ± 0.021	0.480 ± 0.032
E/A ratio	1.554 ± 0.070	1.735 ± 0.117	1.762 ± 0.066	1.698 ± 0.0840	1.645 ± 0.089
MV DecT (ms)	53.83 ± 3.692	62.33 ± 2.883	55.00 ± 3.759	58.00 ± 3.425	53.00 ± 3.162
e′/a′ ratio	1.284 ± 0.158	0.744 ± 0.056 *	1.458 ± 0.155 ^#^	1.249 ± 0.153	0.968 ± 0.032
E/e′	16.48 ± 1.512	24.92 ± 1.114 **	16.31 ± 0.918 ^##^	20.58 ± 0.766	19.74 ± 1.156
LAP * (mmHg)	22.34 ± 1.875	32.79 ± 1.381 **	22.12 ± 1.138 ^##^	27.42 ± 0.949	26.38 ± 1.433
LVOT Vmax (m/s)	0.768 ± 0.037	0.998 ± 0.026 **	0.780 ± 0.029 ^#^	0.768 ± 0.008 ^#^	0.812 ± 0.039
IVCT (ms)	15.67 ± 2.906	18.00 ± 3.777	18,83 ± 3.516	22.83 ± 3.167	16.83 ± 2.272
IVRT (ms)	32.50 ± 1.258	30.33 ± 3.007	32.50 ± 1.057	35.17 ± 1.515	34.00 ± 1.438
LV ET (ms)	87.67 ± 3.116	63.67 ± 1.961 *	101.7 ± 3.703 ^###^	87.83 ± 5.282	80.67 ± 1.745
Tei-index (MPI)	0.558 ± 0.058	0.724 ± 0.051	0.471 ± 0.027 ^#^	0.669 ± 0.055	0.632 ± 0.039
RV E vel. (m/s)	0.332 ± 0.026	0.497 ± 0.044	0.368 ± 0.034	0.383 ± 0.048	0.364 ± 0.027
RV A vel. (m/s)	0.498 ± 0.041	0.628 ± 0.073	0.578 ± 0.033	0.235 ± 0.003 ^#^	0.356 ± 0.074

Parameters measured are as the follows: Left ventricle internal diameter in systole and diastole (LVIDd, LVIDs); left ventricle fractional shortening (FS), ejection fraction (EF); early mitral filling velocity (MV E vel.); late (atrial) filling velocity (MV A vel.); ratio of mitral early/atrial peak velocities (E/A ratio); deceleration time of E wave (MV DecT); ratio of early diastolic mitral annular velocity/late (atrial) diastolic annular velocity (e′/a′ ratio); ratio of early mitral filling velocity/early diastolic mitral annular velocity (E/e′); calculated Left Atrial Pressure (LAP); maximal velocity of left ventricle outflow tract (LVOT Vmax); isovolumic contraction time (IVCT); isovolumic relaxation time (IVRT), left ventricle ejection time (LV ET); Tei-index as myocardial performance index (MPI); right ventricle early filling velocity (RV E vel.); right ventricle late filling velocity (RV A vel.). GotoK rats exhibited differences in E/e′, E wave, e′/a′, LVOT Vmax, LV ET and RV E wave in comparison to the Wistar controls. The abovementioned parameters were significantly improved in the BGP-15 treated GotoK rats, compared to the untreated GotoK animals. Data is expressed as mean ±SEM, *n* = 6/group, Kruskal-Wallis test with Dunn’s post-test; * vs. Wistar (*p* < 0.05); ** vs. Wistar (*p* < 0.01); ^#^ vs. GotoK (*p* < 0.05); ^##^ vs. GotoK (*p* < 0.01); ^###^ vs. GotoK (*p* < 0.001).
